# Generating toxic landscapes: impact on well-being of cotton farmers in Telangana, India

**DOI:** 10.1080/13648470.2017.1317398

**Published:** 2018-06-28

**Authors:** Nanda Kishore Kannuri, Sushrut Jadhav

**Affiliations:** a Indian Institute of Public Health, Hyderabad, India; b Division of Psychiatry, University College London, UK

**Keywords:** Green Revolution, agro-chemicals, cotton cultivation, toxic landscapes, well-being, clinically applied anthropology, India

## Abstract

Existing literature demonstrates agro-chemicals result in physical toxicity and damages human health, flora and fauna. However, little is known about how such ‘toxicity’ relates to mental well-being and social suffering. This paper aims to demonstrate how local, national and international vectors are interlinked to shape social distress among cotton farmers in India. Ethnographic interviews and focus group discussions were conducted in a cotton-growing village of the Warangal district, Telangana state, India. The results advance the concept of counter therapeutic spaces and hypothesise that toxic landscapes emerge through a dynamic interaction between dispersed agencies that interact and reconfigure agricultural spaces into socially toxic places. The paper argues that the disciplines of public health and agriculture suffer from a failure of imagination to forge vital interdisciplinary links that could address farmer suffering. Unpacking local ecologies of farmer suffering offer innovative ways for enhancing mental health policy and interventions in India.

## Agriculture, pesticides and well-being

The majority of India's population (56.6%) is dependent on agriculture and allied occupations for their livelihood (Government of India 2011). Indian agricultural production during the post-independence period fell short of the country's needs. Also, extreme drought in most parts of the country during the mid-1960s forced the Indian government to import grain from the United States (Fitzgerald-Moore and Parai 1996) and promote a Green Revolution (GR) in collaboration with international governments and development agencies (Sebby 2010). This involved the introduction of water management practices, agro-chemicals, mechanisation, high-yielding varieties.^1.^ (HYV) of rice and wheat, and the provision of credits to farmers (Parayil 1992). The GR transformed traditional subsistence-based farming in India into a capital-intensive, modern, surplus-producing agriculture, drastically increasing overall food grain production by 140% over the period 1960–2000 (INSA 2001; Davies 2003).

The spread and application of the GR had an unequal impact on the farming community across the country. The GR technology primarily benefited large farmers who had access to input investment, information, resources, and state support (Sebby 2010). One of the key features of the GR was the the introduction of HYV. The introduced HYV seeds resulted in the reduction of plant height and plant growth duration, photoperiod insensitivity, and greater pest and disease resistance. Despite these changes, HYV depended on large quantities of nitrogenous fertilisers to generate the expected crop outputs. Without these fertilisers, the produce from HYV was considerably less (Shiva 1991).

Approximately four decades following the GR's introduction, the increase in crop yields corresponded with increased fertiliser and pesticide usage (Pimental 1996). However, the long-term application of high quantities of fertiliser led to a decrease in natural soil fertility and a reduction in yields. Narrow genetic-based varieties of HYV of rice and wheat and mono-cropping led to high vulnerability to pests and diseases, while continuous application of pesticides led to resistance among pests. This resulted in excessive dependence on pesticides to limit crop loss (Shiva 1991), resulting in a drastic increase of pesticide use in India: from 154 metric tonnes in 1954 – 88,000 metric tonnes in 2000 (Mathur et al. 2005), an increase of 570% in less than half a century.

The excessive use of pesticides affects human and animal health and causes loss of biodiversity, degradation of the natural ecosystem and irreversible changes in the environment (Khan 2012; Pimental 1996). The collective health impact include: (1) health impairment due to direct or indirect contact, (2) contamination of water, both surface and ground, (3) circulation of pesticide residue in the food chain, (4) resistance among pests, (5) elimination of beneficial organisms, such as insects and birds that are natural predators of the pests and (6) reduction in micro-organisms which maintain soil fertility (Pingali and Roger 1995).

The usage of agrochemicals (fertilisers, herbicides and pesticides) are also known to cause environmental damage. Implementation of the GR has led to the degradation of the environment in multiple ways: (1) monoculture, the cultivating of a single crop variety in all seasons, led to the extinction of the native germplasm and loss of genetic diversity, (2) increased plant vulnerability to pests, (3) long term usage of fertilisers led to increased levels of nitrates and phosphates, reducing oxygen in water, (4) a declining water table, (5) soil erosion, (6) deforestation and (7) exploitation of fossil fuels (Chamala 1990).

It has been suggested that India is witnessing a ‘second GR’ – known as the Gene Revolution – following the introduction of genetically modified Bt cotton (Kumbamu 2006). Introduction of genetically modified (GM) crops adds a new dimension to the issue of toxicity of the landscapes. At a broader level, GM crops raise ethical, environmental and health concerns including resistance and emergence of secondary pests, increasing pesticide usage, decreasing genetic diversity by promoting mono-cropping, escalating seed costs that benefit large farmers and harm to other organisms in nature (Dale, Clarke, and Fontes 2002; Stone 2011).

The outspread of the GR varied over the agro-climatic zones of former Andhra Pradesh (AP) and the recently formed Telangana State.^2.^ The early phase was largely confined to resource-rich coastal regions. In more recent times, farmers from the semi-arid, rain-fed areas adopted GR practices and cultivated high-value commercial crops (Galab, Revathi, and Reddy 2009). Farmers during 1979–1982 and 1998–2002 shifted from cultivating food grains to commercial crops such as oilseeds, sugar cane, chillies and cotton. Similarly, small and marginal farmers shifted from subsistence farming to commercial farming, exposing them to market risks (Bhat et al. 2006).

This prevailing agrarian crisis in the country resulted in farmer suicides. Adverse climatic conditions, failure of the cotton crop and growing debts in the rain-fed cotton-growing regions of AP, Maharashtra and Madhya Pradesh States led to suicides specific to cotton farmers. According to the National Crime Records Bureau (NCRB), the number of suicides in these regions amounted to 68% of the total 284,694 farmer suicides during the 1995–2012 period. NCRB data shows that during 1995–2012, the AP State reported 35,898 farmer suicides. Suicide among farmers was first reported in AP State during the mid-1980s, became persistent during the mid-1990s (Galab, Revathi, and Reddy 2009), and continues to rise (Samdani 2017). Many small and marginal farmers who were affected by agrarian crises resorted to taking their own lives. The suicide rate among Indian farmers was 47% higher than the national average, according to the 2011 census (Baba 2015). More than 300,000 farmers have committed suicide in the country between 1995 and 2014. This translates into approximately one suicide every 30 min (Basu, Das, and Misra 2016).

## Cotton farming in Andhra Pradesh state

After the failure of hybrid varieties owing to their vulnerability to pests, GM Bt Cotton was introduced in AP State around 2002. Farmers rapidly switched to growing Bt cotton amidst competing claims on the effectiveness of Bt cotton. Within a decade, AP state led cotton cultivation in the southern region with 18.79 lakh ha in 2011–2012 (CCI 2013). Bt cotton was aggressively promoted by multinational corporations who projected high financial returns due to higher yields and reduction in pesticide costs (Sainath 2012). Some of the key problems identified in cotton cultivation that led to diminishing yield and distress in AP state are: (1) multiplicity of cotton hybrids, (2) monocropping, (3) extensive use of chemical fertilisers leading to soil imbalance, (4) excessive chemical pesticide, (5) spurious seeds, fertilisers and pesticides and (6) adverse climatic conditions (Gopalakrishnan, Manickam, and Prakash 2007). These factors – along with drought, increasing input costs and debt caused by high interest rates for loans from non-formal credit sources, government policy on subsidies and imports, and the promotion of cotton varieties unsuitable to market conditions, small holdings of the farmers and overdependency on monsoon and insufficient irrigation facilities – drove affected farmers to commit suicide (Cariappa and Cariappa 2004; Ghosh 2005; Kumar 2002; Nagaraj 2008; Parthasarathy and Shameem 1998; Shiv and Visvanathan 1998; Stone 2007; Suri 2006; Samdani 2017).

This paper addresses how the GR and the gene revolution impacted upon the well-being of the cotton farmers in rural areas of India. For analysis, the authors have adopted a broad framework of well-being, which includes individual subjectivity in the context of family, community and society and a range of environmental, geographic, socio-economic and political factors (La Placa, McNaught and Knight 2013).

## Methodology

Ethnographic interviews and two focus group discussions (FGDs) were conducted with male and female cotton farmers from different caste groups including Reddys, Kummari, Goundla, and Kuruma^3.^ communities. Additional interviews with non-qualified medical practitioners popularly, RMPs,^4.^ and mental health professionals in the district (both public and private) focused on health problems of the farming community and their health-seeking behaviour.

Farmers who visited a local pesticide and seed dealer were recruited for the FGDs. Out of the 12 farmers approached, 8 of them expressed willingness to discuss their concerns with the first author. Two declined to participate citing non availability of time and a busy schedule. Participants’ age ranged from 30 to 68 years. The FGD with male farmers – FGD1 – was conducted in an open space near the pesticide shop in the village, during early afternoons when the farmers had finished their work. The discussions lasted approximately two hours.

FGD2 was conducted with women from families who cultivated cotton and also participated in agricultural activities. A total of 8 women (32–50 years), belonging to different caste groups, participated. The women participants were recruited through a snowballing method, with the help of the landlady of the house where the first author resided. FGD2 was conducted in the courtyard of their homes during late mornings when participants were free from household and agriculture activities. The discussions lasted approximately an hour.

Both FGDs broadly covered topics on various domains of agriculture including usage of agrochemicals; impact on the environment; knowledge, attitude and practices around agrochemicals; views on Bt cotton and its effectiveness; health impacts of agrochemicals; help-seeking behaviour and perceived impact of changing landscapes.

Although a similar set of questions guided both the FGDs for men and the FGDs for women, the focus of discussion was different. With men, it was noted that the younger participants were more vocal in expressing their views about agrochemicals and their impact. Women discussed more the changing nature of social relations with the advent of cotton in their village.

All the FGDs and interviews were conducted in Telugu. Data were recorded, transcribed and translated into English followed by thematic analysis. Results are presented under headings with relevant themes that exemplify specific domains of enquiry. All quotations have been transliterated into English to maintain fidelity with the language of study subjects. Reliability was ensured by triangulation of data collected through FGDs and stakeholder interviews, with parallel ethnographic observations during the 12-month period.

## Results

1. Agrochemicals: attitudes, impact on soil and local ecology

While discussing the need for agrochemicals in agriculture, most farmers found it was essential for the production of crops and protection from pests. They felt that the fertilisers make the produce more suitable for market, whether it is food grains, vegetables or cotton. The farmers emphasised the aesthetic demands of buyers in the market as a yardstick for their produce:

‘The buyers are more keen to see how the produce looks. Vegetables should look healthy and gleaming; paddy should look full and dark brown, cotton bolls should look well formed. Unless we please the buyer, we cannot sell our produce. Therefore we are more concerned about the look of the product than the chemicals. If we do not sell after all our hard work, we will starve. We know fertilisers make the product look nice.’ (Participant 1, 38 yrs, male cotton farmer, FGD1;)

Contrary to the populist views, it became evident that farmers were indeed aware of the impact of excessive agrochemicals on nature. They appeared to be remarkably perceptive in identifying sophisticated links between agrochemicals, soil fertility, financial consequences, health risks and local ecology. The impact of the fertilisers and pesticides on the soil, health and environment, is illustrated in the following responses:

‘All these fertilisers have killed the soil. Nothing can grow in these lands now. Only with fertilisers can we grow anything. However, the escalating costs of urea and other fertilisers have broken the back of the farmers. We small farmers cannot apply these many fertilisers now because of the increased costs. Soil got used to these chemicals.’ (Participant 3, 40 yrs, male cotton farmer, FGD1).

‘If a person consumes *mandu*^5.^. for long we all know it has a negative impact on his health. Similarly if soil is fed with fertilisers and pesticides are sprayed continuously, soil will be dead. Nothing will grow there.’ (Participant 1, 38 yrs, male cotton farmer, FGD1)

Commenting on the how pesticides affected food for local birds, a female cotton former commented:

‘The consequence of increased application of pesticides is seen in the village. Many birds which we saw during our childhood are not seen now. House sparrows are vanished. For Dasara festival, it is auspicious to sight Palapitta [Indian Roller] and Nalla Poli Pitta [Black Drongo]. We heard that in cities these birds are caged so that people can go and see them. We have reached to that stage. Birds which feed on grain sometimes are poisoned because of the pesticides and other birds which feed on insects do not have food to eat, as pesticides kill all the insects. There is no doubt that these insects are killed but many other living beings are also killed. Which nobody these days is bothered. Everyone wants their own prosperity.’ (Participant 2, 48 yrs, female cotton farmer, FGD2)

2. Bt cotton: alien and toxic

Discussion on the introduction of Bt cotton elicited varied and contradictory responses. Farmers had different opinions regarding the efficacy of Bt cotton and its impact on the environment. Most mentioned a reduction in the quantity of pesticides and frequency of spraying pesticides was helpful but expensive. They felt that Bt cotton was prone to other pests and justified continuing use of pesticides. Some small farmers fearing bollworm infestation sprayed insecticides in increased quantities, mixed different pesticides in various ratios, often as a preventive measure. However, other pests such as the sucking fly and white insects had increased. This led to a further increase in the use of pesticide. The following quotes illustrate contradictory views on the consequences of Bt cotton crops and pesticide usage:

‘One should agree that Bt cotton changed the pesticide scenario. Earlier we were regularly spraying huge quantities of pesticides. Now it has reduced. The money spent on the quantity of pesticides is decreased. However the new pesticides are more concentrated, can be used in smaller quantities, more powerful, but expensive too.’ (Participant 3, 40 yrs, male cotton farmer, FGD1)

‘Even after Bt the farmers are forced to spend money on pesticides because of other pests. White flies and sucking pests are causing more harm now. Farmers spray pesticides sometimes as preventive measure. They see their neighbours spraying and they do it too even if it is not required. The anxiety of losing crops pushes them to do so.’ (Participant 6, 48 yrs, male cotton farmer, FGD1)

‘I think the power of Bt cotton is reducing. The yield from these is also reducing over the years. Seed companies do this to make more profits by introducing more expensive seeds. Farmers have no other option, but to buy them.’ (Participant 1, 38 yrs, male cotton farmer, FGD1)

Female farmers were of the opinion that biodiversity decreased due to the fact that everyone in the village wanted to cultivate cotton. They lamented that returns from cotton sales were spent to buy other grains, pulses and vegetables. In a series of FGDs, the following responses illustrate gendered notions on the relationship between bio-diversity and cotton cultivation:

‘During olden days there was a saying that it is impossible to name neither the varieties of paddy nor the types of fish in the lakes. All these are gone now. There are not many types of paddy left and so is the case of the fish.’ (Participant 5, 50 yrs, female cotton farmer, FGD2)

‘These days everyone wants to grow cotton. We are forced to buy everything else from shops. Most of the money is spent and we are not sure of the quality of things we buy.’ (Participant 7, 44 yrs, female cotton farmer, FGD2)

‘We never had to buy some things earlier, we used to exchange between neighbours and relatives the things each of us do not grow and do not have. That sharing and exchange is largely disappeared. Everything has to be bought and money has to be paid. There are small shops to sell things now in each corner of the street. We are no different from towns and cities. I think people forgot to give and take things now.’ (Participant 1, 50 yrs, female cotton farmer, FGD2)

Interestingly, for most of the farmers and their families, the concept of Bt cotton meant there was a toxic *(‘visham’*) foreign entity inserted into the cotton plant. This made the cotton ‘impure’. They did not use cotton from the Bt plants to make wicks used to light up lamps for religious worship, suggesting local cultural resistance. One respondent explained:

‘In my family, we do not use Bt cotton for lighting the god's lamp. The wick made of this cotton burns faster with a black soot. It smells also. People say there is something mixed in it. We grow some cotton in the back yard from the seeds we got from our relatives. It is even difficult to get the pure cotton seeds these days.’ (Participant 4, 55 yrs, female cotton farmer FGD2)

Similarly, other informants reported that the BT cotton stalk could not be used for cooking due to higher generation of smoke and its fast burning rate. Some villagers, shepherds by profession, mentioned that they are careful about not allowing their sheep to graze in the Bt Cotton fields as they felt that the grass in those fields was toxic. They said that earlier farmers used to shelter sheep in their farms during the fallow period so that the sheep dung would organically rejuvenate the soil.

Ethnographic notes documented further responses to this theme:

‘We have heard of cattle dying after eating Bt [cotton]. As far I know, there are no instances in our village. But as a protective measure, we do not allow the cattle to graze in the Bt cotton, but it is everywhere… So we graze our sheep and goats in areas further away, where there is grazing land.’ (Male shepherd, 50 yrs, interview notes)

‘No farmer wants to have our sheep shelters in their farms. Everyone uses fertilisers now. Farmers want to grow as much they can from their fields. They are not keeping the land fallow for it to rejuvenate. This I think will eventually deplete all the goodness of the soil.’ (Male shepherd 45 yrs, interview notes)

3. Effectiveness of agrochemicals and alternatives

Farmers’ perception of the effectiveness of agrochemicals was based on the notion, ‘the more the better’. In practice, this translated into an additional quantity of agrochemicals used (in excess of the prescribed quantity), increased number of sprays and pesticides mixed to form a cocktail, and the purchase of more expensive agrochemicals. The effectiveness of agrochemicals was also assessed by the farmers based on their local popularity:

‘Most farmers go by approximate measures. Generally we add a bit more proportions [of pesticides] as we feel it will work better. We have seen paddy, vegetables and cotton crops growing beautifully when applied fertilisers. What else does a farmer want?’ (Participant 1, 38 yrs, male cotton farmer, FGD1)

‘At one point most farmers in the village were convinced that applying potash will make the produce look appealing. So everyone started to apply potash repeatedly. It was also cheap then. But excessive use of potash burnt the soil.’ (Participant 3, 40 yrs, male cotton farmer, FGD1)

Many informants shared their experience of trial and error in order to optimize cotton yield and often viewed themselves as passive agents to explain their collective response towards a seemingly irrational strategy:

‘We farmers are like Sheep. One does something and if it works, all of us do the same. If a farmer benefits from using a particular company's pesticide or fertilisers, all others follow and buy the same things. Sometimes, smaller farmers [borrow] loans to buy products from the same company. (….not opt for other company products…) although they are equally effective and less expensive.’ (Participant 2, 32 yrs, male cotton farmer, FGD1)

‘If as a shopkeeper, I suggest other alternatives, the farmer grows suspicious and might go to another shop to buy. For the fear of losing customers, we also do not try to convince. We try and stock up that particular company's products to cater to the farmers.’ (Male pesticide shop owner, 35 yrs, interview notes)

Whilst discussing alternatives to the practice of excessive usage of agrochemicals, most respondents could not think of any other option. Some mentioned that they had heard about organic farming and vermicomposting.^6.^ Many expressed doubts about the efficiency of these methods. An important concern related to additional burden of labour on both farmers and their families:

‘Labour is in a shortage. Small farmers and their families are forced to do all agricultural activities by themselves.…. extra effort is not an incentive….. it is better to invest the same effort in getting extra income….many of the young men of small farming communities prefer… petty day jobs in the city. In these situations, who will take up organic farming?’ (Participant 2, 32 yrs, male cotton farmer, FGD1)

4. Sources of knowledge

Farmers in the study area were dependent on seed dealers and fellow farmers for information on the right variety, combination, and quantity of pesticides to be used. Peer pressure and a preventive outlook encouraged farmers to apply pesticides and fertilisers even if it was not required. This is revealed through responses from both pesticide shop owners as well as cotton farmers:

‘Farmers come to us for advice. We are in touch with new developments in the field because representatives of the seed and fertiliser companies come and discuss with us. They sometimes organise meetings and they invite us to participate. Since the same company markets the seeds, they know what are the fertilisers required, the quantity, and the time of application. They are more aware of the likely pests, different pesticides, and combinations.’ (Male pesticide shop owner, 40 yrs, interview notes)

‘We trust the pesticide dealer as he is a fellow villager, a farmer himself. So we know what he is using in his farm. We also learn from watching other successful farmers in the village. These days, pesticide and seed companies are setting up demonstration plots in villages and supplying farmers with seeds, fertilisers and pesticides. These plots are closely monitored by the staff of these companies. They pass on this knowhow to the farmer. This helps us to decide on which company seeds, fertilisers and pesticides to use. We see the output practically and get to know details from our fellow villagers.’ (Participant 8, 60 yrs, male cotton farmer, FGD1)

‘These scientists do farming in ideal conditions. They do not know the actual situation in the fields. They are not updated. It is their job. But for us it is our life. The scientist gets paid his salary even though he fails but we can't afford to do that.’ (Participant 5, 30 yrs, male cotton farmer, FGD1)

5. Health impact: signs and symptoms

The farmers seem to be aware that continuous exposure to agrochemicals lead to illness. They reported that, during hot summer and windy seasons, many farmers who spray pesticides experienced dizziness, nausea and itching of the skin. Some of the farmers associated these chemicals with rising instances of cancer among farmers and their families, diabetes, greying of hair, mental instability, lack of physical strength, and youth taking to alcohol. The relationship between chemicals and their health consequences are illustrated through responses that seek to establish direct causality with local concepts of diseases:

‘These chemicals are very powerful. A small capful can kill a person within minutes. If ingested accidentally it will lead to nausea, headaches, blurring of the sight, skin itching. These things keep happening.’ (M8, 60 yrs, male cotton farmer, FGD1)

Several respondents commented on more serious health consequences including cancer, mental illness and diabetes:

‘There are increasing instances of cancer. In the same family, a 22 year old woman had throat cancer and her father had colon cancer. They do not have any [bad] habits, how can you explain this? There are many other cases that people do not disclose.’ (Participant 5, 30 yrs, male cotton farmer, FGD1)

‘I think these chemicals also cause madness. One of the villagers few years ago was spraying pesticides and he fell unconscious. He never recovered completely. The family went to doctors, healers and everyone but there is no change. He is normal but talks to himself and laughs.’ (Participant 8, 60 yrs, male cotton farmer, FGD1)

‘Eating this *mandu kudu* [food laced with chemicals] is leading to a lot of problems like diabetes, greying of hair even among the younger people and many new diseases. The youth these days can't even work as long as we do in the farms. They lack strength. They get tired easily. That is why I think most of the younger generation are drinking too much.’ (Participant 7, 58 yrs, male cotton farmer, FGD1)

6. Safety precautions and the risks to family

Most subjects were aware of risks involved in handling pesticides. However, it was observed that risk perception was not translated into practices that involved safety, protection and safe disposal measures. Protection gear was not worn while mixing and spraying pesticides. The small farmers mixed pesticides at home where they are stored, and carry them in spray tankers to the farm. Sometimes farmers mixed agrochemicals in the fields, and washed the containers in nearby water sources. The empty cans and packets with traces of chemicals were left strewn in the fields or near water sources. It was also observed that the concept of risk was linked to gendered ideas related to risk behaviour. Cultural stereotypes of men being ‘reckless’, women ‘careful and cautious’ influenced their perception of safety and protection measures. Thus, a male cotton farmer illustrated his reasoning:

Many of us are careless when it comes to taking safety precautions though we know about the side-effects. We have been doing this for years and feel nothing will happen to us. If someone tries to be more others make fun of them saying: why you are wrapping yourself like a woman? (Participant 5, 30 yrs, male cotton farmer, FGD1)

Some farmers highlighted that they were not trained in handling pesticides. They followed what others had been doing. Any attempt to implement safety measures was ridiculed. For a small farmer, it was expensive to buy and maintain protective gear and viewed as a worthless investment:

We don't have training on pesticide and fertiliser use. And all these protective gloves, masks are expensive and are not easily available. Small farmers can't spend money on these fancy things and also maintain them. (Participant 2, 32 yrs, male cotton farmer, FGD1)

Ethnographic inquires revealed that the role of women in pesticide application decreased with the advent of newer agrochemicals which required smaller quantities and less water. Earlier, women were largely involved in fetching water, and in preparing the pesticide mix. Application of herbicides reduced the exposure of women to harmful chemicals. As a consequence, their involvement in weeding and exposure to pesticides was reduced. However, they continued to be exposed to such toxins as they are stored at home and during harvesting food grain, vegetables and cotton.

7. Help seeking behaviour

Majority of farmers reported they did not seek medical attention for minor incidents of pesticide poisoning. They explained the impact would subside after some time. However, with the increased awareness, they were concerned about the health effects of chronic exposure to agrochemicals but were unsure about mitigating these effects and deciding when to consult a doctor.

Even if we get exposed accidentally to any chemicals, we immediately wash the area. We do not go to a doctor for these small things. The symptoms like redness of the eyes, dizziness, and skin rashes will go away by themselves. (Participant 8, 60 yrs, male cotton farmer, FGD1.)

Some admitted to a sense of confusion and hypothesised chemical aetiologies for chronic and serious health conditions:

These things are ok, but we do not know what these chemicals are doing to our organs inside the body. As he [another subject in the FGD] said, they all might get accumulated and lead to cancer. These things we do not know until one is very sick and goes to a doctor. (Participant 2, 32 yrs, male cotton farmer, FGD1)

The study site included three unqualified doctors, locally known as RMP doctors, and a qualified Ayurvedic practitioner providing allopathic services.^7.^. Interviews with RMP and mental health professionals from the nearby town indicated broader social determinants linked to health problems and suicides among farmers and explain outward migration.

Many elderly people come to me with body pains. Old age could be one reason but their worries and agony they face is translated into their pains. Their inability to take care of their agricultural lands has led to children moving out of the village. All [of this] impacts on them. I give them some vitamin tablets and an injection for pain relief. This makes them feel better. (RMP 1, 38 yrs, interview notes)

The RMPs offer basic primary health services to the villagers. They refer patients with medical complications to qualified doctors in the nearby town. This comprised a nexus involving referral networks to private diagnostic centres and nursing homes for which they get paid. It was observed that RMPs did not administer any treatment to subjects with pesticide ingestion, either accidental or willful. This is because until 2017 attempted suicide was a punishable offence according to the Indian Penal Code.^8.^ Suicide victims have to be taken to government hospitals as most private hospitals decline to admit them. They explained that:

Most of the common ailments in the village are seasonal. We deal with them. We do not treat any case of pesticide poisoning because it could lead to medico-legal problems for us. It involves dealing with police. It is also risky for us as we can't save the patient. We immediately ask them to rush to the government hospital. (RMP1; RMP2; RMP3; interview notes suggested similar practice amongst all 3 RMPs)

Response from psychiatrists in government and private sectors indicated that suicide amongst cotton farmers was a complex phenomenon interlinked with socio-economic conditions. As suicide is a criminal offence, deliberate self-harm cases are reported as ‘accidents’. There is no follow-up of treatment. This leaves many patients untreated and vulnerable:

There are instances of patients coming in after accidental exposure. Reports of young children accidentally ingesting chemicals are also there. Sometimes deliberate self-harm cases are also brought in as accidental exposure fearing police cases. Most patients who consume pesticides for attempted suicide reach [us] at an advanced stage. They are beyond any chance of revival. I think easy access to pesticides is an important issue. In most of the suicide cases, victims take decisions impulsively. If the access to pesticides is restricted then I presume we can contain the numbers. Suicides among farmers are largely because of the debts and their inability to repay. I do not completely see it as a public mental health problem. (Psychiatrist, 39 yrs, Government hospital, interview notes)

Most suicides are a result of social and financial matters. Farmers commit suicide more because of their inability to clear their debts. (Private psychiatrist, 40 yrs, interview notes)

Notions about mental health problems in the community and the shortage of human resources trained in mental health were important reasons for not seeking mental health services:

‘Most of the villagers ascribe supernatural reasons for [their] mental health problems. Therefore they seek treatment from temples, Babas and Dargahs rather than mental health professionals. The shortage of mental health professional also adds to this. (Psychiatrist, 39 yrs, Government hospital, interview notes)

8. Changing landscapes

Modern agriculture transformed the agricultural landscape of the study village. This transformation resulted from a range of factors such as: cash crops linked to the market economy; the changing aspirations and outlook of the villagers in regards to material conditions; migration; rising alcoholism; as well as unwillingness by the younger generation to pursue agriculture as their occupation. Such factors resulted in major consequences on the social fabric of the village as illustrated below:

Most of their children would have migrated to the nearby cities and towns in search of livelihood. Youth of these days do not prefer to live in village and pursue agriculture. If they are little educated they move to cities like Hyderabad, and if not they go to nearby towns to look for opportunities. (Participant 8, 60 yrs, male cotton farmer, FGD1)

Earlier we used to be satisfied with three meals a day, a decent house and a good crop. Nowadays the needs of these young people are so many. They need all they see in the city – bikes, phones, clothes… They spend a lot of money. (Participant 7, 58 yrs, male cotton farmer, FGD1)

The relationships also changed in the village. Collectiveness is gone. Getting together in the evenings and at festivals is scant. Almost every house has a television and all of them sit in front of it all the time. People who do not have television go to their neighbours house but do not miss these [soap] serials. (Participant 1, 50 yrs, female cotton farmer, FGD2)

Everybody is after money now. When people meet, they enquire about cotton price of the day. This has become the centre of our lives… one wouldn't talk about it if the prices are stable. They [prices] dance up and down leading to worries. (Participant 7, 58 yrs, male cotton farmer, FGD1)

Who will take care of the elders left behind in the village? The older generation is used to a particular kind of life which is close to the land they own. Most of us do not want to go to these cities and live. Children being away, decreasing [cotton] yields, inability to manage fields because of shortage of labour. We all have health problems. (Participant 4 68 yrs, male cotton farmer, FGD1)

The advent of the market economy into the village also changed its social structure. While the GR benefited a few large farms, most medium-sized and small-sized farms experienced fluctuating returns, as much depended on timely availability of accessible loans, favourable climatic conditions and market prices. At the time of the field work, the land value in and around the study village had increased more than 100 times in some places as there was a proposal to merge the village with the city municipal corporation. Farmers who were interviewed shared invaluable insights detailed on land prices, social disparity, and consequences to their well-being:

The land value is increasing day by day. Our children want to sell it and move on to the city. Some of us are doing it, but many of us do not want to - as land is the only thing we have. There is a proposal that our village will be a part of the greater city. Land prices and demand will go up even further. We will be under more pressure to sell. All this will lead to conflict within the families. (Participant 7, 58 yrs, male cotton farmer, FGD1)

Big farmers can sell some of the land for huge money and invest money somewhere to make more profits. We small farmers with small bits of land will lose everything if we sell our lands. That is why although there is demand we are not willing to sell. We can never buy the land back. (Participant 2, 48 yrs, female cotton farmer, FGD2)

Some rich upper caste farmers in the village want the village to be merged with the corporation. They have land. They can sell and make huge amounts of money. Many farmers who belong to backward castes and who have little land, and a majority of the villagers who belong to scheduled castes, oppose this as we do not benefit in anyway. Rather, we will lose as we will have to pay more taxes to the corporation. (Participant 8, 60 yrs, male cotton farmer, FGD1)

## Discussion

The application of science and technology in agriculture has been posited as a global solution for human development (PRM 33 1978; Alain de et al. 2005). This approach influenced agricultural policies adopted by the Government of India during the post-independence era (Sebby 2010). Following independence, India had to adopt the GR in the context of several constraints including a shortfall of food grains, impending famines, Malthusian projections of population explosion, and political pressure from the United States of America (Shiva 1991). Responding to rapid globalisation, India's agricultural policy aligned with neo-liberal policies and promoted cash crops, pushing farmers prematurely into a perilous global market (Suri 2006). Such policies also led to the creation of novel ecosystems causing a massive and damaging ecological impact (Hobbs, Higgs, and Harris 2009).

In this paper, the authors extend these developments to the Gene Revolution – as it involved a rapid spread of Bt cotton cultivation, leading to destabilisation of local ecologies. Many farmers in dry and arid areas adopted Bt cotton, leading to extraction of excessive ground water, the high application of agrochemicals, and mono-cropping. The creation of such new ecologies resulted in biotic and abiotic stress, brought about a shifting relationship between plants, animals, birds, microorganisms, land and humans. Taken together, the GR and the Gene revolution led to an adverse impact on the cultural landscape and well-being of India's rural population.

The study findings in this paper suggest that changing landscapes of a cotton farming village impact on the well-being of people – and document why and how distress among cotton farmers in turn scape agricultural spaces.

Rural life in India is popularly considered as unpolluted, green and pure; and idealised as containing a harmonious social bond amongst villagers (Nandy 2001). Indeed, this idea resonates with the notion that such landscapes promote health and well-being, and are ‘therapeutic’ in nature (Gesler 1992; Rose 2012). In earlier reports, the authors have documented how the toxicity of markets, agrarian practices, natural disasters, human-animal conflicts, and competition over mining resource – mediated through structural violence, generate mental health concerns and challenges that are poorly addressed by both mental health professionals and policy (Jadhav et al. 2015). The concept of ‘counter-therapeutic’ spaces resulting from asymmetric interactions between people, the environment, and institutions governing them was proposed to explain the generation of socially ‘toxic’ landscapes (ibid. page 13).

In this paper, agricultural landscapes are shown to be actively counter-therapeutic and differ from a more passive notion of ‘non-therapeutic’ landscapes (Jadhav and Barua 2012). The results of this study also suggest that mental distress experienced by cotton farmers cannot be separated from local ecologies which generate suffering, and risk being glossed over by popular ‘global mental health models’ and policies (Lancet Global Mental Health Group 2007).

**Figure 1.**
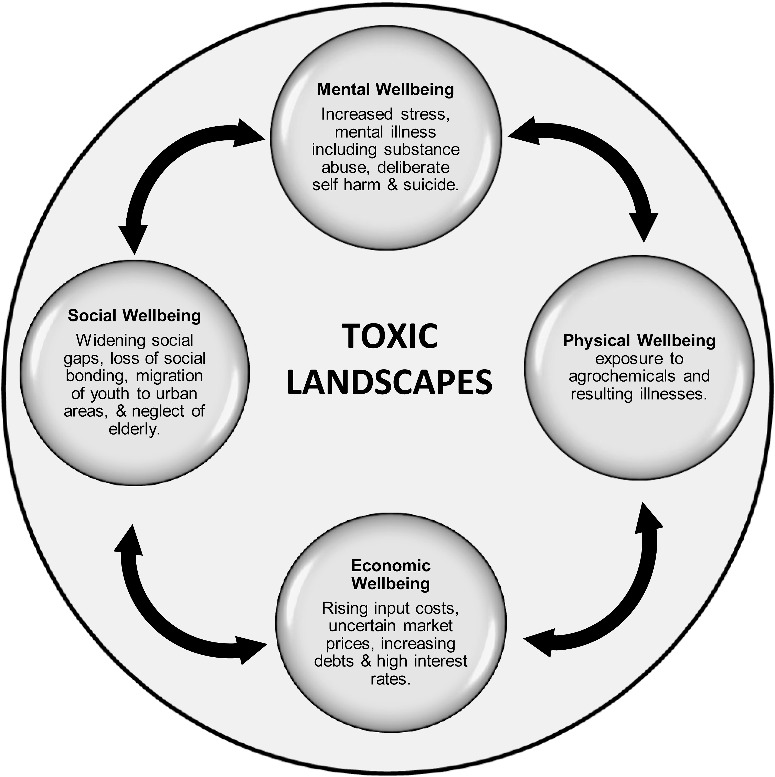
Toxic landscapes: mental, physical, economic and social components.

Based on findings documented in this paper, the authors hypothesise that cotton farming villages in India are being rapidly transformed into socially ‘toxic landscapes’ that intertwine and interdigitate with cotton farmers’ distress. Such socially ‘toxic’ landscapes do not result from physical consequences alone. Landscapes operate, shape and impact upon human well-being through multiple, complex cultural, psychological, and economic vectors. Such processes cannot be simply understood as linear, cause and effect outcomes (Casey 2009). They result from a dynamic interaction between a range of stakeholders in the form of dispersed agencies that both constitute and reconfigure agricultural spaces, and lead to the emergence of socially ‘toxic landscapes’. The distress that cotton farmers experience is, therefore, an embodiment of ‘toxicity’ which is simultaneously physical, social, and psychological – both in nature and consequence. This is illustrated in Figure 1.

This hypothesis is in keeping with earlier research among socially isolated agricultural farmers in the USA and argues for an appreciation of agency as an emergent outcome of ‘network collectives that include people, institutions, and technologies’ (Trauger 2009). Although Trauger's analysis, based largely on Latour's ‘actor-network theory’ points to the role of leadership and a handful of leading actors influencing the shape the direction and outcome of such discourse – the present study does not align with Trauger's suggested conclusion. This is more likely due to differing objectives and theoretical disciplinary frames adopted by researchers of the two studies, and therefore does not permit direct comparison of interpretation/s.

## Agricultural distress and the failure of imagination

It is evident that cotton farmers experience significant distress as a result of crop failure. Although this paper does not systematically detail the nature and severity of such distress, it provides sufficient evidence to confirm the failure of State services responsible for cotton farming policy and intervention.

The authors argue that the disciplines of public health and agriculture in India suffer from a failure of imagination to make vital inter-disciplinary connections that might address farmer suffering. The substantial body of literature cited at the start of this paper identifies the specific role of agricultural policies, scientists and market forces in shaping cotton farmer's distress – yet the role of public health professionals especially mental health, is limited to epidemiological studies and policy statements, lamenting the absence of more ‘psychological autopsies’, and demanding a ‘greater need for State role’ (Rao et al. 2017). Indeed, the data from this study documents a range of psychiatric problems experienced by cotton farmers including increased stress, substance abuse, deliberate self-harm, and suicide, together with challenges in utilising existing State mental health care. As space and authors’ expertise limit a fuller interpretation and discussion of the role of Agricultural and State policies, this section will focus on unpacking the role of India's mental health professionals in addressing the psychological distress of cotton farmers. More specifically, this section examines why and how mental health professionals have failed in their imagination, and what might be possible to reinvigorate a crucial link between psychiatry and local culture.

Mental health training and practice in India is chiefly a watered down version of Western psychiatry (Jain and Jadhav 2008). This dominant practice is not without resistance against dominant biomedical models. Alternative formulations exist but seldom find their voices incorporated into official policy (Bayetti, Jadhav, and Deshpande 2017). If they do, they fail at the point of delivery and outcome as what constitutes ‘evidence’ rarely incorporate local concerns over what is defined as ‘recovery’ in the Indian context (Bayetti, Jadhav, and Jain 2016). Indeed, existing state endorsed clinical practices continue to edit out local suffering – critical for mental health intervention and outcome (Jain and Jadhav 2009). The reasons for carving out presenting patients’ ‘psychopathology’ as disembodied culture free findings which privilege universal phenomenological ‘form’ over ‘culture-specific content’ have been extensively discussed (Kleinman 1987; Littlewood 1990). However, the more challenging and complex nexus between ‘local’ and ‘global forces’ with ‘content’ and ‘context’ bound nature of cotton farmers’ mental suffering remains unexamined. Existing pedagogy and institutional dynamics in low-income nations, together with more global pressures to conform and comply with international (proxy for more ‘western Euro-American’) pressures demand and stifle local, imaginative ruptures on the part of mental health professionals’ response to relate and engage with local suffering. Such local patient-professional relationships are vital to forge empathic bonds between mental health professionals with those who suffer. They also carry tremendous potential for generating clinical ethnographies which in turn may shape the evidence to generate local and national policy (Jadhav and Jain 2012). Although the much-proclaimed phrase of ‘scaling up resources’ (Patel et al. 2007; Eaton et al. 2011) to meet with an acute shortage of mental health professionals is indeed relevant, it popularises a top-down approach to address the vast mental health burden in India. In doing so, such policy sidesteps the actual scantily documented reality of clinical – cultural interaction with local communities, and fails to validate the suffering of majority rural Indian population for whom mental distress is one of several consequences resulting from existing agricultural failure. The crucial role of ethnographic insights including collaboration with social scientists, toward a cross-disciplinary engagement, is missing. This paper hypothesises that the social position of State professionals suits their professional and personal careers at the expense of continuing distress of local communities – a disincentive for re-thinking culturally responsive solutions to seemingly global problems. Indeed, existing widely prevalent Eurocentric theory and practice suits the needs of dominant classes in India (Nandy 1983). However, to demand that mental health professionals and agricultural scientists alone bear the burden of addressing farmers’ social suffering is equally untenable. In this context, more recent clinically applied anthropological techniques such as the ‘cultural formulation’ (CF) approach, may well equip mental health clinicians to contextualise and document mental suffering and its relationship with their local landscapes. The Bloomsbury Cultural Formulation Interview (BCFI 2010) is one of several different CF's developed by clinicians in diverse settings to address local concerns (.Lewis-Fernández et al. 2014). The BCFI is both an approach and technique which involves a sustained and continuing cultural dialogue between patient and clinician, a method that seeks to enhance therapeutic engagement and enrich clinical assessment for patients of all social backgrounds, and aimed at eliciting a structured narrative account of suffering, deploying the metaphor of ‘culture’ (Napier et al. 2014). Although the (BCFI) was developed initially in London, it has now been piloted for use in rural India (Jadhav and Jain 2012; Jadhav et al. 2015). The authors of this paper do not claim that any specific clinical ‘instrument’ such as the BCFI can indeed fix the problem of ‘culture’ (Kleinman and Benson 2006). However, the proposed technique offers one of several ways to reconceptualise and widen the lens through which cotton farmers could be better engaged and understood by mental health professionals when they develop mental distress. In brief, cultural clinical approaches like the BCFI allow clinicians to generate local clinical ethnographies and challenge existing clinical vocabularies of suffering. Such cultural formulation interview approaches also offer potential to establish what troubles people most, capture personal explanations of their distress, detail local cultural context, plot vectors and spatiality of sites that generate suffering, and identify disciplines that need to be engaged toward ameliorating the plight of people who place their trust in State services. The findings documented in this paper suggest that distress develops in the context of local ecologies which require cultural sensitivity and more crucially, a social-ethical response on the part of mental health clinicians. This may well offer the potential for intervention on ‘their’, not ‘our’ terms. This is in contrast to deploying a conventional psychiatric approach that medicalises farmers’ distress which in turn generates biomedical categories empty of social meaning. Additionally, the development of systematic accounts of such clinical ethnographies could create a crucial ‘database’ and hypotheses for future intervention based research. This suggested framework also offers potential to powerfully impact on State Health and Agricultural policies including implications for future mental health training texts and service delivery guidelines. Such approaches could also contribute to the shaping of a bottom-up policy that will entail coordinated effort from several identified disciplines within each context, to bear upon a specific individual or community's local ecology of suffering, across cultures.

## Conclusion

In conclusion, this paper demonstrates how local, national and international vectors may interlink to shape local distress among cotton farmers in Telangana State, India. The paper advances existing notions of counter therapeutic spaces and hypotheses that ‘toxic’ landscapes are an emerging property resulting from a dynamic interaction between dispersed agencies that reconfigure agricultural spaces into socially damaging places. To mitigate the impact of changing landscapes and farmers’ distress, a multi-pronged approach is necessary. This paper proposes a cross-disciplinary collaboration between cotton farmers, agrarian and social scientists, and health professionals – as an essential and initial step towards evolving socially relevant interventions and outcome for cotton farmers in India. Indeed such approaches may help identify linkages and potential bridges amongst identified stakeholders to transform physical, psychological and social toxicity into more ‘healing’ landscapes. Until then, the ‘gift relationship’ between Indian cotton farmers with policymakers, agricultural and health professionals, and consumers of cotton will remain ethically and culturally asymmetric (Mauss 1966).

## Notes

1. High-yielding varieties are defined as short, high-yield producing early-maturing types in comparison to traditional varieties, under intensive agricultural practices including ensured irrigation and application of chemical fertilizers (Parayil 1992).

2. Telanaga region of Andhra Pradesh was formed into Telangana State on 2 June 2014. The field area of the study, Warangal district is a part of Telangana state.

3. The study village consisted of different caste groups who took up cotton cultivation for their livelihood. Reddys are a landed caste group traditionally part of the farming community. Other caste groups like Kummari, Goundla and Kuruma belong to lower caste groups.

4. RMP is an acronym for Registered Medical Practitioners. The RMP system was discontinued in India many years ago. For most of the rural population in India, these unqualified personnel are the first contact point for any health care.

5. Mandu in Telugu language refers to alcohol, medicine, and pesticide in different contexts.

6. Organic farming emphasises holistic soil and crop management using natural methods and techniques. Vermicomposting is a method of composting organic material using earthworms.

7. It is common for many Ayurvedic practitioners to provide biomedical care.

8. According to article 309 of the Indian Penal Code (IPC), attempting to commit suicide is an offence, punishable with one year's imprisonment or a fine, or both. Government of India decriminalized attempt to suicide through The Mental Health Care Act 2017. http://www.prsindia.org/uploads/media/Mental%20Health/Mental%20Healthcare%20Act,%202017.pdf
